# Suppressive Effects of *Clerodendrum volubile* P Beauv. [Labiatae] Methanolic Extract and Its Fractions on Type 2 Diabetes and Its Complications

**DOI:** 10.3389/fphar.2018.00008

**Published:** 2018-02-01

**Authors:** Ochuko L. Erukainure, Rahman M. Hafizur, Nurul Kabir, M. Iqbal Choudhary, Olubunmi Atolani, Priyanka Banerjee, Robert Preissner, Chika I. Chukwuma, Aliyu Muhammad, Eric O. Amonsou, Md. Shahidul Islam

**Affiliations:** ^1^Nutrition and Toxicology Division, Federal Institute of Industrial Research Oshodi, Lagos, Nigeria; ^2^H.E.J. Research Institute of Chemistry, International Center for Chemical and Biological Sciences, University of Karachi, Karachi, Pakistan; ^3^Department of Biochemistry, School of Life Sciences, University of KwaZulu-Natal, Westville Campus, Durban, South Africa; ^4^Dr. Panjwani Center for Molecular Medicine and Drug Research, International Center for Chemical and Biological Sciences, University of Karachi, Karachi, Pakistan; ^5^Faculty of Science, Institute of Biological Sciences, University of Malaya, Kuala Lumpur, Malaysia; ^6^Structural Bioinformatics Group, Institute for Physiology, Charité – University Medicine Berlin, Berlin, Germany; ^7^Department of Chemistry, University of Ilorin, Ilorin, Nigeria; ^8^Department of Food Technology, Durban University of Technology, Steve Biko Campus, Durban, South Africa; ^9^Department of Pharmacology, University of the Free State, Bloemfontein, South Africa; ^10^Department of Biochemistry, Ahmadu Bello University, Zaria, Nigeria

**Keywords:** biochanin, *Clerodendrum volubile*, immunohistochemistry, type 2 diabetes, 5,7,4’-trimethoxykaempferol

## Abstract

Type 2 diabetes is the most prominent of all diabetes types, contributing to global morbidity and mortality. Availability and cost of treatment with little or no side effect especially in developing countries, remains a huge burden. This has led to the search of affordable alternative therapies especially from medicinal plants. In this study, the antidiabetic effect of the methanolic extract, dichloromethane (DCM), butanol (BuOH) and aqueous fractions of *Clerodendrum volubile* leaves were investigated in type 2 diabetic rats for their effect on glucose homeostasis, serum insulin level and hepatic biomarkers, lipid profile, pancreatic redox balance and Ca^2+^ levels, and β-cell distribution and function. The DCM was further fractionated to isolate the active compounds, biochanin and 5,7,4′-trimethoxykaempferol. They were investigated for their toxicity and ADMET properties, α-glucosidase and angiotensin I converting enzyme (ACE) inhibitory activities *in silico*. There were significant (*p* < 0.05) decrease in blood glucose, cholesterol, LDL-C, vLDL-C, triglyceride, AST and ALT levels in all treated groups, with DCM fraction showing the best activity. All treated rats showed significantly (*p* < 0.05) improved anti-oxidative activities. Treatment with the DCM fraction led to significant (*p* < 0.05) increased serum insulin and pancreatic Ca^2+^ levels, as well as improved β-cell distribution and function. DCM fraction also showed improved glucose tolerance. DCM fraction dose-dependently inhibited ACE activity. The toxicity class of the isolated compounds was predicted to be 5. They were also predicted to be potent inhibitors of cytochrome P (CYPs) 1A2, 2D6 and 3A4. They docked well with α-glucosidase and ACE. These results indicate the therapeutic potential of the plant against type 2 diabetes, with the DCM fraction being the most potent which may be attributed to the isolated flavones. It further suggests antihypertensive potentials of the DCM fraction. However, inhibition of CYPs by the flavones may suggest caution in usage with other prescribed drugs metabolized by these enzymes.

## Introduction

The treatment and management of diabetes mellitus (DM) remains a global health challenge, with developing countries being the most affected. DM is a chronic disease characterized by increased blood glucose due to the pancreatic β cells unable to produce insulin as in the case of type 1 diabetes (T1D), or inability of the body to effectively utilize the insulin produced as in the case of type 2 diabetes (T2D) (Erukainure et al., 2013). T2D is recognized as the most prevalent (>90%) of the two types, accounting for 12% of global total health expenditure and five million deaths in 2015 (International Diabetes Federation [IDF], 2015). It mostly occurs as a result of insulin resistance and β-cell dysfunction, leading to chronic hyperglycemia (Prentki and Nolan, 2006; Cerf, 2013). Increased hyperglycemia triggers excessive production of free radicals, resulting in oxidative stress (Tiwari et al., 2013), which is responsible for the pathogenesis of most diabetic micro- and macro-vascular complications (Constantino et al., 2013).

There is a growing interest in the use of medicinal plants for the treatment as well as management of T2D (Patel et al., 2012; Chikezie et al., 2015). These plants have been employed in folkloric medicine from time immemorial. This has been attributed to their phytochemical constituents with reported anti-oxidative and anti-diabetic activities (Saeed et al., 2012). Moreover, they are readily available and affordable, with lesser side effects as compared to most synthetic antidiabetic drugs. Amongst such plants is *Clerodendrum volubile* of the *Clerodendrum* genus.

Often regarded as magic leaf, *C. volubile* is among the common food ingredients in Southern Nigeria, and also employed in the folkloric treatment of diabetes, ulcer, arthritis, rheumatism, and dropsy (Burkill, 1985; Erukainure et al., 2014). We reported in our previous studies, the anti-oxidative potential of an isolated iridoid glycoside from the leaves in brain and hepatic tissues (Erukainure et al., 2014). We also correlated the antidiabetic potential of the ethyl acetate fraction to the immunomodulatory activity of isolated protocatechuic acid (Erukainure et al., 2017a). We have shown the ability of dietary fatty acids from the leaves to arrest proliferation in human breast cancer cells (Erukainure et al., 2016b). The ability of the leaves extracts to inhibit to α-amylase, α-glucosidase and angiotensin-1 converting enzyme has been reported recently (Adefegha and Oboh, 2016). Studies have also demonstrated the ability of the chemical fractions and fatty acids of the flower and stem to modulate phagocytic oxidative burst (Erukainure et al., 2016a, 2017c). Despite these studies, there is still a dearth of information on the effect of the leaves on T2D complications and other compounds that may contribute to its acclaimed antidiabetic properties.

To the best of our knowledge, the isolation and characterization of two flavones, 5,7,4′-trimethoxykaempferol and 4′-methoxy-5,7-dihydroxy isoflavone (biochanin) from the dichloromethane fraction of *C. volubile* leaves methanolic extract has not been reported previously. Thus, the present study also reports the anti-diabetic effects of the chemical fractions of *C. volubile* leaves in type 2 diabetic rats followed by bioactivity-guided isolation, and structure elucidation of the active compounds. The anti-hypertensive, oral toxicity and drug-like activities of the flavones were also studied *in silico*.

## Materials and Methods

### Instrumentation

^1^H- and ^13^C-NMR spectra (400 and 100 MHz, respectively) and two-dimensional correlation spectra COSY, NOSEY, HMQC, and HMBC, were recorded on a Bruker AV-400 spectrometer in CDCl_3_. δ (ppm) values were used for reporting chemical shifts. Hitachi UV-3200 spectrophotometer was used for obtaining the UV spectra. Thin layer chromatography (TLC) was done on pre-coated silica gel 60 F_254_ plates (E. Merck, 0.25 mm), and detected under UV light (254 nm) and by spraying with ceric sulfate reagent. The EI-MS (Electron Ionization Mass Spectrometry) spectrum was measured on a JEOL-MSRoute HX 110 mass spectrometer using the standard positive mode.

### Plant Materials

Fresh *C. volubile* leaves purchased from local farmers at Ifon, Ondo State, Nigeria, were identified and authenticated by Dr. Henry Akinbosun of the Department of Botany, University of Benin, Benin City, Nigeria. It was assigned the voucher number, UBH_C284_ and thereafter deposited at the herbarium. The leaves were air-dried, pulverized to fine powder, and stored in air-tight containers till further analysis.

### Extraction and Fractionation

Four and half (4.5) kg of the blended sample was subjected to methanol (MeOH) extraction at room temperature. The resulting extract was concentrated *in vacuo* using Buchi Rotavapor (Model: R-300, Buchi, Switzerland) to yield 450 g crude extract. One hundred gram (100 g) of the crude extract was subsequently dissolved in distilled water and further subjected to liquid–liquid fractionation using gradient polarity solvents in the order: *n*-hexane (Hex), dichloromethane (DCM), ethyl acetate (EtOAc) and *n* – butanol (BuOH). The fractions were concentrated *in vacuo*, while the aqueous residue was freeze dried.

### Enzyme Inhibitory Assay

The methanolic crude extract and fractions were evaluated for their antidiabetic potentials by assaying their inhibitory activities against α-glucosidase (Oboh and Ademosun, 2011; Ramsay et al., 2012). Based on the results, the crude extract, DCM, BuOH, and aqueous fractions were selected for *in vivo* studies.

### Animals

Forty-two male albino rats of Wistar strain weighing about 180–200 g from Animal Resource Facility of International Center for Chemical and Biological Sciences (ICCBS), University of Karachi, Karachi, Pakistan, were used for the study. They were acclimatized on standard rat pellet chows for 1 week, with water provided *ad libitum* under standard laboratory conditions of natural photo period of 12-h light-dark cycle. All animal studies were carried out under the approval and guidelines of the biological ethical committee of the International Center for Chemical and Biological Sciences (ICCBS), University of Karachi, Karachi, Pakistan, in accordance to the Declaration of Helsinki. The protocol approval number is 2015-0020.

### Induction of Type 2 Diabetes

Type 2 diabetes was induced using the streptozotocin-nicotinamide model (Erukainure et al., 2017a; Noor et al., 2017). The rats were first intraperitoneally injected with nicotinamide (120 mg/kg bw), then 15 min later with streptozotocin (60 mg/kg bw) intravenously. The control groups were injected with the required volume of citrate buffer and normal saline only.

After 2 weeks, rats were considered diabetic with blood glucose level > 190 – 200 mg/dl and used for the study.

### Acute Antidiabetic Studies

Extract and fractions (200 or 400 mg/kg bw) or saline were administered orally to overnight-fasted diabetic rats. Glibenclamide (GB) was used as a positive control. Blood was taken immediately before (0-h) and at 1-, 2-, and 3-h after extract/GB treatment. Based on the results obtained, 400 mg/kg bw was selected as the effective dose.

### Experimental Design

The rats were divided into seven groups, each consisting of six animals.

Group 1 – Normal rats + Pelletized rat chows
Group 2 – Diabetic (Untreated)
Group 3 – Diabetic + *C. volubile* crude extract (400 mg/kg bw)
Group 4 – Diabetic + *C. volubile* DCM fraction (400 mg/kg bw)
Group 5 – Diabetic + *C. volubile* BuOH fraction (400 mg/kg bw)
Group 6 – Diabetic + *C. volubile* aqueous fraction (400 mg/kg bw)
GB – Diabetic + Glibenclamide (5 mg/kg bw)

The extract dose was selected based on previous studies (Erukainure et al., 2017a). Food and water intake, as well as body weight were monitored daily for each rat. While their blood glucose levels were monitored on weekly basis with a glucometer (Accu-Chek). Treatment lasted for 3 weeks. At the end of the experiment, the rats were subjected to overnight fasting and anesthetized with sodium thiopental (60 mg/kg), then humanely sacrificed.

### Serum Preparation

Blood was collected via cardiac puncture and centrifuged at 3000 rpm for 10 min. Serum was separated, aliquoted and stored at -80°C for further biochemical assays. The ultra-sensitive rat insulin ELISA kit (Crystal chemicals, Downers Grove, IL, United States) was used in determining the serum insulin level.

β-Cell function was quantified via the homeostatic model assessment (HOMA)-CIGMA software.

### Determination of Hypolipidemic Activities

Commercial assay kits from Randox^®^ Laboratories, United Kingdom, was used in the determination of serum total cholesterol (TC), triglyceride (TG), and high density lipoprotein (HDL) by colorimetric method according to manufacturer’s protocol. The concentration of low-density lipoprotein (LDL) cholesterol and very low-density lipoprotein (vLDL) cholesterol were calculated by the following formula (Friedewald et al., 1972).

### Determination of Hepatic and Renal Function Enzymes

Serum hepatic and renal biomarkers which covers for aspartate transaminase (AST), alanine transaminase (ALT) and urea were analyzed using commercial kits from Randox^®^ Laboratories, United Kingdom, according to the manufacturer’s protocol.

### Preparation of Pancreatic Tissue Homogenate

Pancreatic organs collected from the sacrificed rats, were washed in distilled water to remove blood. They were chopped, homogenized in phosphate buffer (20 mM; pH 6.6) and centrifuged at 10,000 rpm for 15 min at 4°C. The supernatant was collected, and stored at -20°C for subsequent analysis.

The protein contents of the tissue homogenates were determined by Lowry’s method using bovine serum albumin (BSA) as standard (Lowry et al., 1951).

### Determination of Oxidative Stress Parameters

The serum and pancreatic homogenates were assayed for reduced glutathione (GSH) level (Ellman, 1959), catalase and superoxide dismutase (SOD) activities (Chance and Maehly, 1955; Kakkar et al., 1984), and lipid peroxidation (LPO) (Chowdhury and Soulsby, 2002).

### Determination of Calcium Ion

The calcium ion levels of the pancreatic tissue homogenates were quantified using the Cobas kits and Roche/Hitachi 912 automatic analyzer according to manufacturer’s protocols, with absorbance read at 450 nm.

### Immunohistochemistry

Pancreases were fixed in neutral buffered formalin solution for overnight and embedded in paraplast. Each piece of pancreatic tissue was serially sectioned (5 μm) throughout its length. Six sections in different planes of pancreatic tissues from each group were triple stained for insulin, glucagon and nuclei as previously described (Hafizur et al., 2011, 2012). The fluorescent images were visualized in DAPI, fluorescein isothiocyanate and TxRed channels using Nikon TE2000E fluorescent microscope equipped with a Nikon DS-2MBWc camera. NIS-Elements image analysis software AR 3.0 (Nikon) was used in attaining the images. The images were processed with Adobe Photoshop CS2.

### Oral Glucose Tolerance Test with DCM Fraction in Diabetic Rats

Based on the *in vivo* studies, the DCM fraction was used for oral glucose tolerance test (OGTT). Twenty four type 2 diabetic rats were divided into four groups consisting of six animals each as shown below:

Group 1: Distilled water
Group 2: 400 mg/kg DCM fraction
Group 3: 400 mg/kg crude extract
GB: Glibenclamide (5 mg/kg) treated rats

They were fasted overnight before the test. Groups 2 and 3 were administered crude extract and DCM fraction, respectively, 30 min prior to 3 g/kg glucose administration. Rats (GB) were treated with 5 mg/kg glibenclamide 30 min prior glucose load and used as a positive control. Blood glucose levels of all rats were measured at -30, 0, 30, 60, and 120 min after administration.

### Angiotensin 1 Converting Enzyme (ACE) Inhibition Assay

The ability of DCM fraction to inhibit ACE activity was determined spectrophotometrically by estimating the amount of hippuric acid released from hippuryl-histidyl-leucine substrate upon ACE catalysis (Oboh et al., 2013).

### Isolation of Compounds from DCM Fraction

The DCM fraction was subjected to further fractionation using a gravity column chromatography loaded with silica gel. The column was eluted with mixtures of Hex and DCM (95:5) in increasing order of polarity up to 80:20. Elutes were collected and their purity ascertained by subjecting to thin-layer chromatography on pre-coated silica gel 60 F_254_ sheets and viewed under UV light (254 nm) after spraying with ceric sulfate reagent.

### *In Silico* ADME and Oral Toxicity Prediction of Isolated Compounds

*In silico* prediction of ADME properties and oral toxicity vis-à-vis qualitative structure activity relationship (QSAR) and virtual molecular structure activity relationship studies (SARs) of the isolated compounds were carried out using SwissADME (Daina et al., 2017) and ProTOX (Drwal et al., 2014).

### *In Silico* Prediction of Molecular Targets of Isolated Compounds

The molecular targets for the isolated compounds were predicted using mode of action (MOD) function of the SuperNatural II database (Banerjee et al., 2014). A total of 10 molecular targets were predicted, only prediction above 80 percent accuracy was considered for further validation.

### Computational Docking Studies

The isolated compounds were docked against crystal structure of Human Angiotensin Converting Enzyme (PDB ID: 1O86) and Crystal structure of the N-terminal Subunit of Human Maltase-Glucoamylase Enzyme (PDB ID: 2QMJ) using the Gold version 5.2. The structures of biochanin (PubChem CID: 5280373) and 5,7,4′-trimethoxykaempferol were obtained from NCBI Pubchem database. All the protein molecules were prepared by removing water molecules and hydrogen atoms added. The bioactive conformation of the ligand was used to validate conformation and also define the binding site of the protein (Kitamura et al., 2008) respectively. GOLDScore scoring functions were used to perform all molecular docking runs, while other parameters were used as default. During the docking process a maximum of 300 different conformations was considered for the ligands. The top ten conformers based on highest binding score were used for visual inspections and further analysis. PyMOL was used to visually inspect the docked conformations and the one which had the highest ligand–receptor interactions was selected for further validation. For the Human Maltase-Glucoamylase Enzyme docking, a crystal structure of the protein bound with Acarbose (PDB code: 2QMJ) was used as the receptor molecule. Binding site was defined by removing the acarbose with the radius of 10 Å. The original ligand was docked into the protein molecule in order to reproduce the original interactions observed in the crystal complex as well as to design the standard molecular docking protocol.

### Statistical Analysis

Results were expressed as mean ± Standard Deviation (SD). Differences among groups were analyzed by one-way analysis of variance (ANOVA) using Statistical Package for Social Sciences (SPSS) software, SPSS Inc., Chicago, IL, United States, Standard version 10.0.1. To compare data within group, paired *t-*test (2-tailed) was performed. *P*-values < 0.05 were considered statistically significant for differences in mean using the least of significance difference.

## Results

**FIGURE 1:**
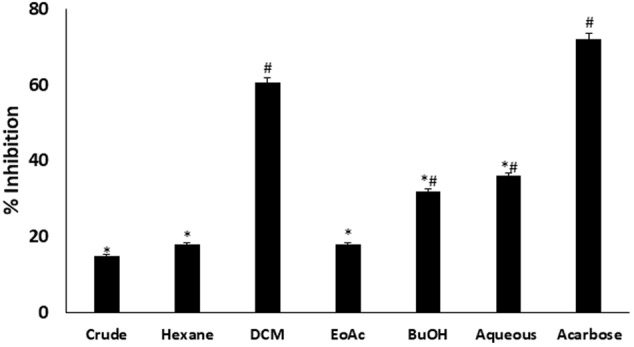
Inhibitory effect of *C. volubile* methanolic extract and fractions on α-glucosidase activity. Data = mean ± *SD*; *n* = 3. ^∗^Statistically significant (*p* < 0.05) compared to Acarbose; ^#^statistically significant (*p* < 0.05) compared to crude extract. Crude, crude extract; Hexane, hexane fraction; DCM, dichloromethane fraction; EoAC, ethyl acetate fraction; BuOH, butanol fraction; Aqueous, aqueous fraction.

*In vitro* α-glucosidase inhibitory activity revealed that DCM fraction significantly (*p* < 0.05) inhibited α-glucosidase activity as depicted in **Figure 1**. BuOH and aqueous fractions also showed significant (*p* < 0.05) inhibitory activities. The inhibition by DCM fraction was comparable to acarbose, a standard inhibitor for α-glucosidase.

During the acute experimental period (3-h), little or no changes in blood glucose level were observed in non-diabetic control rats, and untreated diabetic rats (**Supplementary Figure S1**). Interestingly, after oral administration to diabetic rats, MeOH, DCM, BuOH, and aqueous fractions of *Clerodendrum volubile* showed different degree of blood glucose lowering effects. MeOH significantly decreased the blood glucose levels in a dose-dependent manner, with a significant (*p* < 0.05) reduction at 2-h after treatment with 200 mg/kg bw. No further improvement was observed after this time period. In sharp contrast, treatment with 400 mg/kg bw (MeOH) decreased the blood glucose level dramatically at 2-h, with similar results observed at 3-h after oral administration. In the case of DCM fraction, significant (*p* < 0.05) reduced blood glucose level was attained at 1-h with the dose of 200 mg/kg bw, and more significant reductions at 2- and 3-h. Treatment with 400 mg/kg bw showed more pronounced effect on blood glucose lowering effects. In the case of BuOH fraction, treatment with 200 mg/kg bw showed little or no effects. However, treatment with 400 mg/kg bw showed significant (*p* < 0.05) reduction in blood glucose level at 2-h. No further improvement was observed after this time period. For the aqueous fraction, both doses showed significant (*p* < 0.05) reduction in blood glucose level at 2-h. while the standard drug significantly (*p* < 0.05) lowered the blood glucose in a time-dependent manner.

**FIGURE 2:**
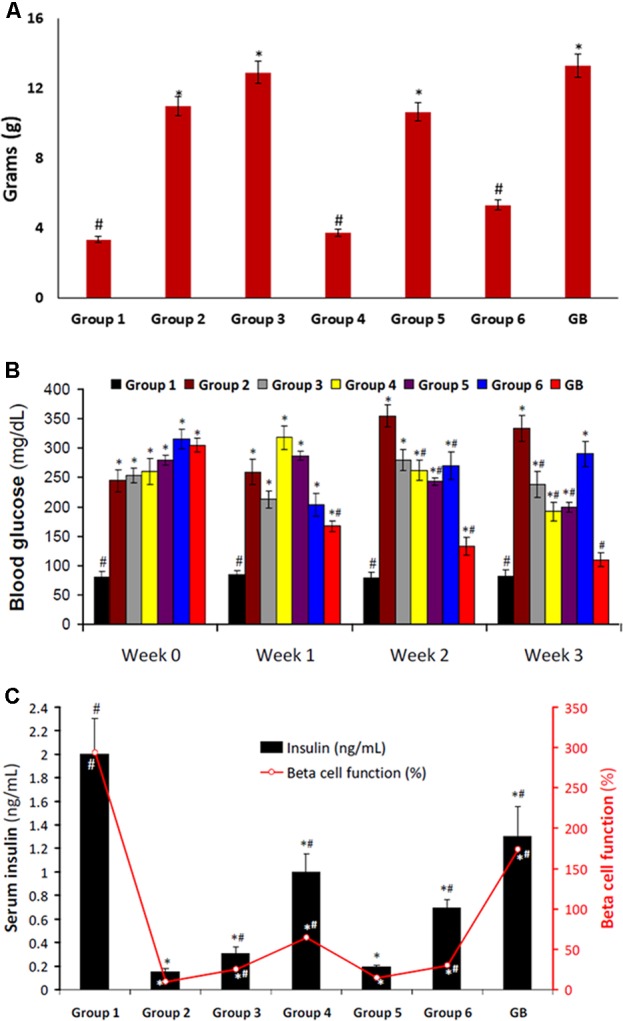
**(A)** Body weight gain of experimental groups. Data = mean ± *SD*; *n* = 6. ^#^Statistically significant (*p* < 0.05) compared to group 2; ^∗^statistically significant (*p* < 0.05) compared to group 1. Group 1: normal control; Group 2: diabetic control; Group 3: diabetic rats + crude extract; Group 4: diabetic rats + dichloromethane fraction; Group 5: diabetic rats + butanol fraction; Group 6: diabetic rats + aqueous fraction; GB: diabetic rats + glibenclamide. **(B)** Blood glucose level of experimental groups. Data = mean ± *SD*; *n* = 6. ^#^Statistically significant (*p* < 0.05) compared to group 2; ^∗^statistically significant (*p* < 0.05) compared to group 1. Group 1: normal control; Group 2: diabetic control; Group 3: diabetic rats + crude extract; Group 4: diabetic rats + dichloromethane fraction; Group 5: diabetic rats + butanol fraction; Group 6: diabetic rats + aqueous fraction; GB: diabetic rats + glibenclamide. **(C)** Serum Insulin level and β-cell function of experimental groups. Data = mean ± *SD*; *n* = 6. ^#^Statistically significant (*p* < 0.05) compared to group 2; ^∗^statistically significant (*p* < 0.05) compared to group 1. Group 1: normal control; Group 2: diabetic control; Group 3: diabetic rats + crude extract; Group 4: diabetic rats + dichloromethane fraction; Group 5: diabetic rats + butanol fraction; Group 6: diabetic rats + aqueous fraction; GB: diabetic rats + glibenclamide.

Induction of T2D led to significant (*p* < 0.05) weight gain as observed in group 2 (**Figure 2A**). Only rats treated with DCM fraction showed significant (*p* < 0.05) weight reduction. The others showed little or no reduction. Glibenclamide treated group also showed body weight gain.

Induction of T2D also led to significant (*p* < 0.05) increased blood glucose level, and decreased serum insulin level and β-cell function as depicted in **Figures 2B,C**, respectively. These were significantly (*p* < 0.05) reversed in rats treated with DCM fraction. BuOH fraction-treated rats also showed significant glucose reduction with concomitant increased serum insulin level. Although the crude extract and aqueous showed non-significant reduction of blood glucose level, however, serum insulin level was significantly increased. β-Cell function was dramatically decreased in the control diabetic rats. However, β-cell function was increased significantly in the crude extract, dichloromethane and aqueous fractions treated rats. The standard drug glibenclamide also improved serum insulin and β-cell function significantly.

**FIGURE 3:**
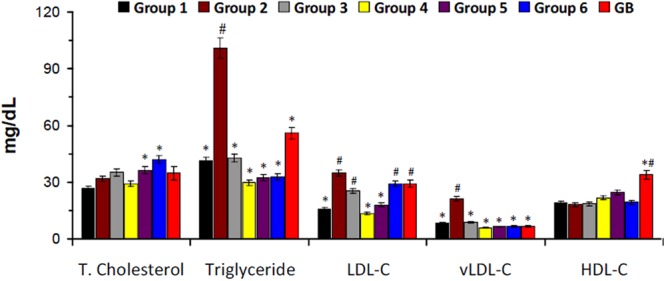
Serum lipid profile of experimental groups. Data = mean ± *SD*; *n* = 6. ^∗^Statistically significant (*p* < 0.05) compared to group 2; ^#^statistically significant (*p* < 0.05) compared to group 1. Group 1: normal control; Group 2: diabetic control; Group 3: diabetic rats + crude extract; Group 4: diabetic rats + dichloromethane fraction; Group 5: diabetic rats + butanol fraction; Group 6: diabetic rats + aqueous fraction; GB: diabetic rats + glibenclamide.

There was a slight increase in serum total cholesterol level in the untreated diabetic rats (group 2) as depicted in **Figure 3**. However, the levels of triglyceride, LDL-C, and vLDL-C were significantly higher in untreated diabetic rats. Treatment with DCM and BuOH fractions caused significant (*p* < 0.05) decrease of their levels. Significant (*p* < 0.05) decreased triglyceride and vLDL-C levels were also observed in groups treated with the crude extract and aqueous fraction. There was no significant difference in the HDL-C levels in the crude extract or its fraction-treated groups. The positive control glibenclamide significantly lowered total cholesterol, triglyceride, LDL-C, and vLDL-C levels and elevated HDL-C level.

**FIGURE 4:**
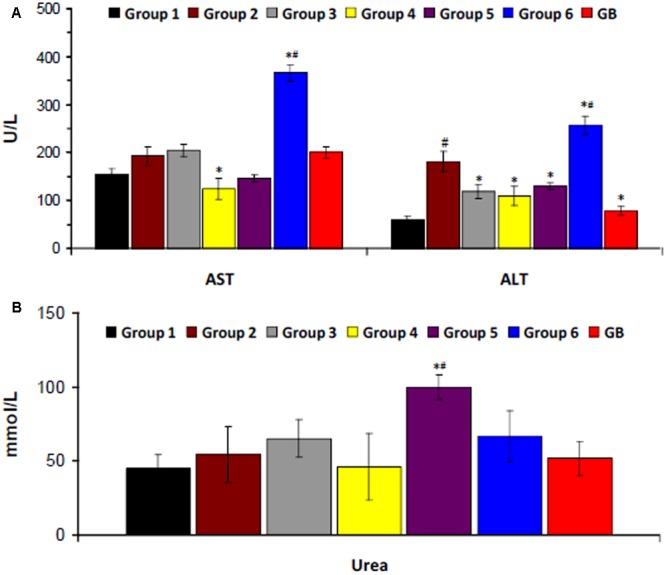
Serum **(A)** hepatic and **(B)** renal biomarkers of experimental groups. Data = mean ±*SD*; *n* = 6. ^∗^Statistically significant (*p* < 0.05) compared to group 2; ^#^statistically significant (*p* < 0.05) compared to group 1. Group 1: normal control; Group 2: diabetic control; Group 3: diabetic rats + crude extract; Group 4: diabetic rats + dichloromethane fraction; Group 5: diabetic rats + butanol fraction; Group 6: diabetic rats + aqueous fraction; GB: diabetic rats + glibenclamide.

There was a significant increase in serum ALT level on induction of T2D as shown in **Figure 4A**. Serum AST level was also increased but was not significant. Treatment with the crude extract, DCM and BuOH fractions significantly (*p* < 0.05) decreased ALT level. DCM fraction also caused a significant (*p* < 0.05) decrease in AST level. Treatment with aqueous fraction significantly (*p* < 0.05) increased both enzyme levels. Induction of T2D led to little or no significant increase in serum urea level. However, significantly higher (*p* < 0.05) levels were observed in rats treated with BuOH and aqueous fractions (**Figure 4B**).

There was significant (*p* < 0.05) decrease in pancreatic GSH level, SOD and catalase activities as depicted in **Table 1**. There was no significant difference in serum GSH level and catalase activity. MDA levels were significantly (*p* < 0.05) increased in both serum and pancreatic tissues. These were significantly (*p* < 0.05) reversed in the treated groups.

**Table 1:** Antioxidant activities in experimental groups.

Groups	GSH (mol/L)		SOD (U/mg protein)		Catalase (U/mg protein)		MDA (mol/L)	
	Serum	Pancreas	Serum	Pancreas	Serum	Pancreas	Serum	Pancreas
Group 1	0.57 ± 0.01	91.82 ± 5.08^∗^	21.03 ± 1.40^∗^	40.09 ± 0.99^∗^	55.22 ± 1.00	121.91 ± 9.45^∗^	211.86 ± 8.99^∗^	314.97 ± 10.84^∗^
Group 2	0.49 ± 0.03	78.27 ± 6.99^#^	16.13 ± 0.99^#^	27.21 ± 0.57^#^	49.46 ± 1.05	74.65 ± 2.08^#^	276.84 ± 9.04^#^	1011.30 ± 18.35^#^
Group 3	0.53 ± 0.01	85.05 ± 9.54	17.39 ± 2.88^#^	31.57 ± 1.86^#^	60.11 ± 3.59^∗^	101.73 ± 7.53^∗^	218.93 ± 10.38^∗^	861.58 ± 14.59^∗#^
Group 4	0.55 ± 0.02	88.11 ± 5.03	21.30 ± 2.99^∗^	35.92 ± 0.84^∗^	72.00 ± 3.99^∗#^	104.32 ± 8.02^∗^	234.46 ± 20.92^∗^	649.72 ± 20.33^∗#^
Group 5	0.59 ± 0.07	94.16 ± 5.11^∗^	20.29 ± 1.72^∗^	30.58 ± 1.05^#^	46.76 ± 3.89	96.68 ± 3.05^∗^	211.86 ± 10.99^∗^	475.99 ± 10.38^∗#^
Group 6	0.55 ± 0.03	87.47 ± 5.90	16.98 ± 1.45^#^	30.51 ± 0.78^#^	55.02 ± 1.02	109.32 ± 4.06^∗^	220.34 ± 12.87^∗^	890.44 ± 21.30^∗#^

*Data = mean +*SD*; *n* = 6. ^#^Statistically significant (*p* < 0.05) compared to group 2; ^∗^statistically significant (*p* < 0.05) compared to group 1. Group 1: normal control; Group 2: diabetic control; Group 3: diabetic rats + crude extract; Group 4: diabetic rats + dichloromethane fraction; Group 5: diabetic rats + butanol fraction; Group 6: diabetic rats + aqueous fraction.*

**FIGURE 5:**
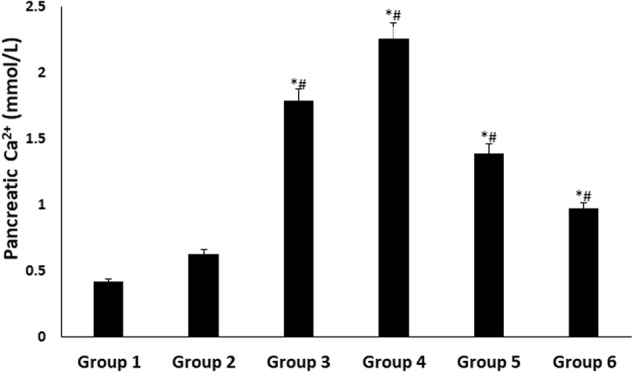
Pancreatic Ca^2+^ level of experimental groups. Data = mean ± *SD*; *n* = 6. ^∗^Statistically significant (*p* < 0.05) compared to group 2; ^#^statistically significant (*p* < 0.05) compared to group 1. Group 1: normal control; Group 2: diabetic control; Group 3: diabetic rats + crude extract; Group 4: diabetic rats + dichloromethane fraction; Group 5: diabetic rats + butanol fraction; Group 6: diabetic rats + aqueous fraction.

There were significant (*p* < 0.05) increases in pancreatic Ca^2+^ levels in all treated groups, with DCM fraction showing the highest level as shown in **Figure 5**.

**FIGURE 6:**
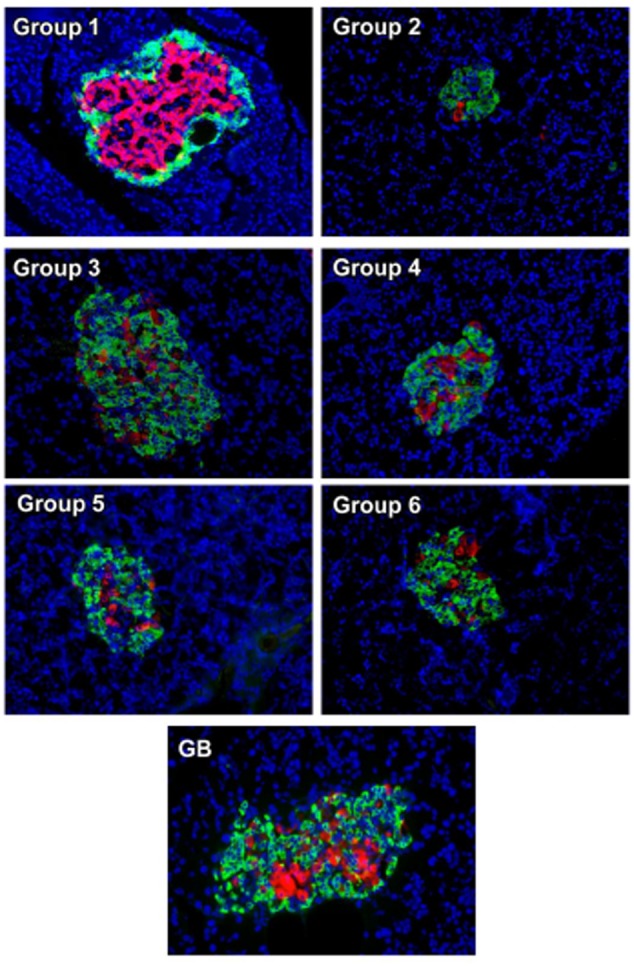
Immunohistochemical analysis of insulin (*red*) and glucagon (*green*) in pancreatic islets of experimental groups. Nuclei are counter-stained with DAPI (*blue*). Magnification ×20. Group 1: normal control; Group 2: diabetic control; Group 3: diabetic rats + crude extract; Group 4: diabetic rats + dichloromethane fraction; Group 5: diabetic rats + butanol fraction; Group 6: diabetic rats + aqueous fraction; GB: diabetic rats + glibenclamide

Immunohistochemistry analysis of pancreas revealed an even distribution of α- and β-cells throughout the pancreatic islets of normal rats (group 1), with the β-cells in abundance and more concentrated in the islets core while the α-cells in the periphery of β-cells as depicted in **Figure 6**. The β-cells were significantly deteriorated in the untreated diabetic rats (group 2) with increased number of α-cells compared to the normal rat. However, treatment with the crude extract and fractions led to an increment in the number and size of the β-cells. This was more pronounced in the crude extract (group 3) and DCM (group 4) treated rats. Glibenclamide treatment also significantly modulated β-cells size as well number. Predominant β-cells hypertrophy was observed in the glibenclamide-treated diabetic rats as shown in GB group.

**FIGURE 7:**
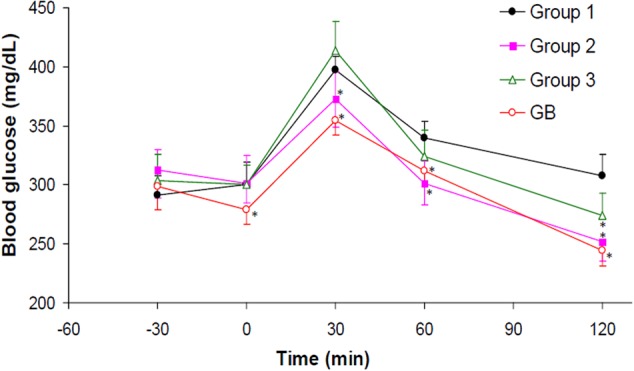
Oral Glucose Tolerance Test (OGTT) of experimental groups. Data = mean ±*SD*; *n* = 6. ^∗^Values are significantly (*p* < 0.05) different compared to group 1. Group 1: diabetic control (distilled water); Group 2: diabetic rats + crude extract; Group 3: diabetic rats + dichloromethane fraction; GB: diabetic rats + glibenclamide.

OGTT assay revealed an attained peak level for all groups at 30 min after glucose administration as shown in **Figure 7**. The crude extract treated rats (group 3) showed significant (*p* < 0.05) improved glucose tolerance compared to the untreated diabetic rats (group 1). Administration of DCM led to significant reduction in glucose intolerance in diabetic rats (group 2). Glibenclamide significantly improved the glucose tolerance in all the time points compared to the untreated diabetic rats.

**FIGURE 8:**
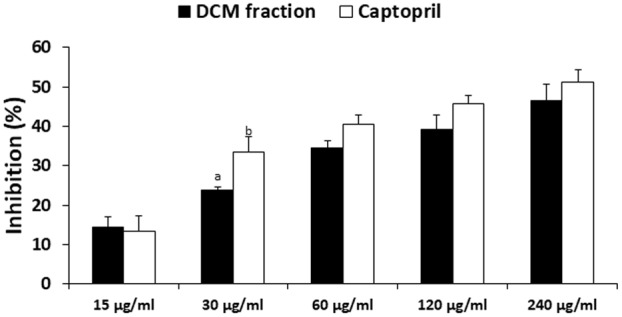
Angiotensin 1 converting enzyme (ACE) inhibitory activity of DCM fraction of *C. volubile* methanolic extract. Data = mean ±*SD*; ^ab^Values are significantly (*p* < 0.05) different from each other.

DCM fraction showed significant (*p* < 0.05) and dose-dependent inhibitory activity on ACE, which compared favorably to the standard drug used as evident by the IC_50_ values (**Figure 8**).

**FIGURE 9:**
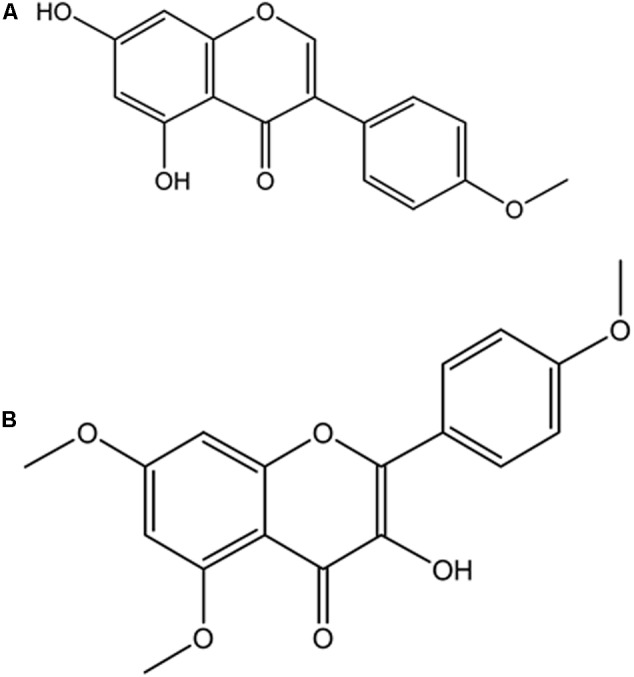
Isolated compounds from DCM fraction of *C. volubile* methanolic extract. **(A)** 4’-methoxj’-5,7-dihydroxy isonnvone (Biochanin); **(B)** 5,7,4’-trimethoxykaempferol.

4′-methoxy-5,7-dihydroxy isoflavone (Biochanin) was obtained from elution with Hex and DCM (85:15) and shown in **Figure 9A**. EIMS m/z 284 [M+H]^+^, 284 (100), 241 (27), 236 (14), 152 (19), 132 (41), 71 (31). ^1^H-NMR (500 MHz, CH_3_COCH_3_, δ, ppm) δ 13.0 (1H, s, OH), 8.01 (1H, s, H-2′), 7.53 (2H, d, H-2′,6′), 7.12 (2H, d, H-3′,5′), 7.10 (1H, d, H-8), 6.64 (1H, d, H-6), 3.90 (3H, s, OCH_3_). The data were compared with literature (Du et al., 2006; Fokialakis et al., 2012).

5,7,4′-trimethoxykaempferol was also obtained from elution with Hex and DCM (82:7.5) and shown in **Figure 9B**. EIMS m/z 314 [M-CH_2_]^+^, 314 (2.5), 302 (3.6), 300 (100), 284 (6.9), 168 (43.2), 133 (15.6), 69 (18) and 44 (72.3). ^1^H-NMR (400 MHz, CH_3_COCH_3_, δ, ppm) δ 12.86 (1H, s, OH), 7.99-8.03 (2H, dd, *J* = 8.8 Hz, H-2′,6′), 7.09-7.13 (2H, dd, *J* = 8.8 Hz, H-3′,5′), 6.65 (2H, s, H-6, H-8), 3.90 (9H, s, 5,7,4′ OCH_3_).

Based on the predictions both compounds biochanin and 5,7,4′-trimethoxykaempferol were classified under the toxicity class, V (**Table 2**), which corresponds to non-toxic class. Their GI absorption was predicted to be high. They were both predicted to be inhibitors of CYP1A2, CYP2D6, and CYP3A4. Biochanin was predicted to be able to cross the blood brain barrier (BBB).

**Table 2:** Predicted toxicity and drug likeness of isolated flavones.

Parameters	Biochanin	5,7,4′-trimethoxykaempferol
GI absorption	High	High
BBB permeate	Yes	No
P-gp substrate	No	No
CYP1A2 inhibitor	Yes	Yes
CYP2C19 inhibitor	No	No
CYP2C9 inhibitor	Yes	No
CYP2D6 inhibitor	Yes	Yes
CYP3A4 inhibitor	Yes	Yes
Log Kp (skin permeation)	–6.66 cm/s	–5.91 cm/s
Oral LD_50_ (rats)	3919 mg/Kg	2500 mg/Kg
Toxicity class	5	5

From the molecular docking results, both compounds, 5,7,4′-trimethoxykaempferol and Biochanin bonded to the active site of the ACE (**Supplementary Figure S2A**). From the ligand–receptor interaction, 5,7,4′-trimethoxykaempferol interacts with the active site of the ACE, forming hydrogen bond with the residues HIS 387, TYR 523, LYS 511. Similarly, Biochanin interacts with the residues HIS 383, GLU 411, HIS 387, TYR 523 (**Supplementary Figure S2B**). These interactions are similar to that observed with the original ligand Lisinopril. The information collected from the docking experiment of the two compounds correlated with the results obtained from the *in vitro* ACE inhibition assay.

Also from the molecular docking results, both compounds, 5,7,4′-trimethoxykaempferol and biochanin bonded to the active site of the receptor molecule (**Supplementary Figure S3A**). Based on the ligand–receptor interaction (**Supplementary Figure S3B**), it can be seen that 5,7,4′-trimethoxykaempferol and biochanin interacted with the enzyme forming bonds with residues ASP 203, ARG 526, PHE 450 and residues ARG 526, TRP 406, ASP 443, HIS 600, respectively. The presence of the hydroxyl/carboxyl groups correspond to the hydrogen bond interactions in the protein active site. In the case of Biochanin (**Supplementary Figure S3B (Right)**), one of the hydroxyl groups corresponds with the hydrogen bond donor to the binding site residue ASP 443, whereas carboxyl group, acted as a hydrogen bond donor to the residue ARG 526. The hydroxyl group of 5,7,4′-trimethoxykaempferol (**Supplementary Figure S3B (Left)**), acted as a hydrogen bond donor to the residue ASP 203 and carboxyl group to the residue ARG 526.

## Discussion

The cost of diabetes treatment is of tremendous concern, especially in developing countries, battling with poor economic growth and other non-communicable diseases. This is responsible for the high diabetic mortality rate in Africa, despite the low occurrence of diabetes compared to the developed world (WHO, 2016). This has led to the search and/or development of affordable alternative medicine from plant source for the treatment and management of diabetes. The use of medicinal plants for healing is part of most African traditions, which is gaining much attention. In this study, we investigated the antidiabetic properties of *C. volubile leaves* and its fractions as well as the compounds that may be responsible for the activity.

*In vitro* inhibition of intestinal α-glucosidase has been demonstrated as a means of screening antidiabetic properties of natural products and developed drugs (Ademiluyi and Oboh, 2012; Erukainure et al., 2017a). Its inhibition causes delay and prolong carbohydrate digestion, thus reducing the rate of glucose absorption (Sun et al., 2015). In consequence, the significant inhibition by the DCM, BuOH and aqueous fractions of *C. volubile* portrays an antidiabetic potential.

To select the best effective dose, we performed acute blood glucose lowering effects of MeOH, DCM, BuOH and aqueous fractions of *C. volubile* on diabetic rats. The acute blood glucose lowering data suggests that treatment with 400 mg/kg bw are more effective, Therefore, 400 mg/kg bw was utilized as the dose for the chronic experiments.

The relationship between obesity and T2D is well documented and has been attributed to progressive decline in insulin secretion leading to concomitant rise in insulin resistance (Golay and Ybarra, 2005). Insulin resistance is the major metabolic abnormality in T2D (Monzillo and Hamdy, 2003). If left unchecked, it will lead to pancreatic β-cell dysfunction thereby causing chronic hyperglycemia and dyslipidemia (Adiels et al., 2008) normally found in the non-obese type 2 diabetic conditions. This corresponds to the increased weight gain (**Figure 2A**), high blood glucose level (**Figure 2B**), depleted serum insulin level and β-cell function (**Figure 2C**) in the untreated diabetic rats (group 2). These were significantly (*p* < 0.05) reversed to near normal notably in diabetic rats treated with DCM fractions (group 4) and GB treated rats, indicating an antidiabetic effect which can be attributed to the synergetic effect of the isolated compounds: biochanin and 5,7,4′-trimethoxykaempferol. Their strong binding affinity and molecular interaction on docking with α-glucosidase (**Supplementary Figures S3A,B**) indicates a strong inhibitory effect which corroborates the high inhibitory activity of the fraction on the carbohydrate catabolic enzyme (**Figure 1**). The impaired glucose intolerance by DCM fraction (**Figure 7**) further portrays an improved insulin secretion, sensitivity and β-cell function (Stumvoll et al., 2000; Yeckel et al., 2004). Thus, portraying an antidiabetic property of the fraction. This corresponds to our previous report on the ability of the ethyl acetate fraction of *C. volubile* methanolic extract to reduce blood glucose level and improve insulin secretion and β-cell function in type 2 diabetic rats (Erukainure et al., 2017a).

The elevated levels of serum cholesterol, triglyceride, LDL-C and vLDL-C as well as decreased HDL-C level in the untreated diabetic rats (group 2) indicates an occurrence of dyslipidemia (**Figure 3**). Insulin resistance alters lipid metabolism in T2D, rendering the lipoproteins pathogenic which has been implicated in the progression of T2D complications (Vijayaraghavan, 2010; Erukainure et al., 2013). This alteration also corresponds to the increased weight gain in the untreated diabetic rats (**Figure 2A**) and can be attributed to the depleted serum insulin level and β-cell function (**Figures 2B,C**). The depleted levels of serum cholesterol, triglyceride, LDL-C and vLDL-C after treatment with the crude extract and fractions therefore indicate a therapeutic anti-dyslipidemic effect.

The depleted GSH level as well as decreased SOD and catalase activities (**Table 1**) in the serum and pancreatic tissues of untreated diabetic rats (group 2) indicates an occurrence of oxidative stress. The role of oxidative stress in the progression and pathogenesis of T2D and its complications has been attributed to the extremely low level of antioxidants in the pancreatic β-cells (Robertson and Harmon, 2007). The levels of SOD and catalase in pancreatic tissue are 50 and 1%, respectively, compared to that of hepatic tissue (Pi et al., 2010). The decreased catalase activity implies an increased production of hydroxyl radical (^⋅^OH) from accumulated H_2_O_2_ due to the depleted enzyme level (Kanias and Acker, 2010). OH then attacks the membrane lipid thereby initiating a peroxidative chain reaction, with MDA and 4-Hydroxynonenal (4-HNE) as the usual end-products (Aslan et al., 2000; Kanias and Acker, 2010). This corresponds to the high MDA level in the untreated diabetic rats (group 2), indicating an occurrence of lipid peroxidation. The reversed GSH and MDA levels as well increased SOD and catalase activities therefore portray an antioxidative protective effect. The high activity in the DCM fraction treated diabetic rats can be attributed to the isolated flavonoids (**Figure 9**). The antioxidative activities of flavonoids are well documented and have been attributed to their fifteen-carbon skeleton consisting of two benzene rings connected by a heterocyclic pyrane ring (Kumar and Pandey, 2013; Vinayagam and Xu, 2015).

Hepatotoxicity and nephrotoxicity have been reported as major complications of T2D owing to insulin resistance and oxidative damage (Vozarova et al., 2002). They are characterized by increased serum levels of hepatic and renal enzymes due to inflammation of the tissues (Erukainure et al., 2015). The increased levels in the diabetic rats (group 2) portrays incidences of hepatotoxicity and nephrotoxicity. The decreased levels after treatment with the crude extract and fractions indicates a protective effect in diabetics and compared favorably with GB treated group. However, the increased AST and ALT levels in diabetic rats treated with the aqueous fraction (group 6) indicates a hepatotoxic effect (**Figure 4**).

Elevated pancreatic Ca^2+^ level has been associated with increased insulin secretion via exocytosis of insulin granules (Rorsman et al., 2012). Ca^2+^ alterations may impair insulin signal transduction, thereby contributing to peripheral insulin resistance (Pittas et al., 2007). The elevated levels in the treated diabetic rats (**Figure 5**), particularly group 4 corroborates the elevated serum insulin levels (**Figure 2C**). This may be attributed to an increased ATP: ADP ratio, causing depolarization of the pancreatic β-cell due to potassium efflux (Shukla et al., 2012). This triggers the opening voltage – calcium channels, allowing an influx of Ca^2+^ into the cell (Pittas et al., 2007; Shukla et al., 2012). Flavonoids have been reported for their ability to open the voltage – gated calcium channels (Kim et al., 2004; Saponara et al., 2008; Scholz et al., 2010), which may be responsible for the increased pancreatic Ca^2+^ levels in diabetic rats treated with DCM fraction.

The uneven distribution of α- and β-cells (**Figure 6**), with the β-cells less in pancreatic tissues of untreated diabetic rats (group 2) corroborates the depleted serum insulin level and pancreatic β-cell function as well as increased blood glucose level (**Figures 2B,C**). The deteriorated β-cells indicates a truncated secretion of insulin typical in T2D. The inappropriate increased α-cells portrays an increased α-cell function and concomitant secretion of glucagon, leading to hyperglycemia (Hafizur et al., 2011, 2017; Godoy-Matos, 2014). Increased hyperglycemia has been implicated in the progression as well as induction and pathogenesis of oxidative stress in type 2 diabetes and may be responsible for the depleted antioxidant activities (**Table 1**) (Constantino et al., 2013; Tiwari et al., 2013). The increased β-cells and evenly distributed α-cells in the treated groups depict an improved β-cell function and hypoglycemic activity, respectively, which also compares favorably with the GB treated rats. Thereby corroborating the increased serum insulin and reduced blood glucose levels (**Figures 2B,C**), as well as improved antioxidant activities (**Table 1**).

Prevalence of the co-existence of diabetes and hypertension has been reported, with 75% of diabetic patients hypertensive (Kaplan, 1987; Grossman and Grossman, 2017). This has been attributed to insulin resistance induced stimulation of the angiotensin 1 receptor (Tuck et al., 2004). Stimulation of the angiotensin 1 receptor activates the angiotensin 1 converting enzyme (ACE), thereby causing an elevated level of angiotensin II which is vasoconstrictive (Oboh et al., 2017). The dose-dependent inhibition of ACE by the DCM fraction (**Figure 8**), therefore indicates a protective effect against hypertension. Several studies have reported the anti-hypertensive properties of flavonoids (Loizzo et al., 2007; Guerrero et al., 2012; Shafaei et al., 2016), which is also reflected in the strong binding affinity and molecular interactions of the isolated compounds (biochanin and 5,7,4′-trimethoxykaempferol) on docking with the crystal structure of Human Angiotensin Converting Enzyme (PDB ID: 1O86) (**Supplementary Figures S2A,B**). This correlates with the increased pancreatic β-cell and serum insulin level as ACE inhibitors have been shown to be not just therapeutic against hypertension but also reduces the risk of type 2 diabetes and its complications (Abuissa et al., 2005; Ramos-Nino and Blumen, 2009).

The toxicity and safety of medicinal plants remains a huge concern to health practitioners particularly modulation of cytochrome P450 enzyme family (CYP) and P-glycoprotein (P-gp). CYPs are the most recognized enzymes involved in drug metabolism, thus their modulation can have therapeutic consequences (Ondieki et al., 2017). The predicted inhibition of CYP1A2, CYP2C9, CYP2D6, and CYP3A4 by biochanin and 5,7,4′-trimethoxykaempferol (**Table 1**), therefore suggests an influential role in drug metabolism. CYP1A2 is involved in the metabolism of caffeine and has been reported in type 2 diabetes patients on caffeine therapy and/or consumes coffee (Urry et al., 2016). Its predicted inhibition by the compounds indicates a low metabolism of caffeine when co-administered, thereby leading to high concentration of caffeine with toxic consequence (high-dose effect). Likewise, co-administration of the compounds with antidiabetic drugs metabolized by the other CYPs (CYP2D6 and CYP3A4) will also elicit an over dose effect of the drugs. Inhibition of CYP2C9 by biochanin may be advantageous, as CYP2C9 metabolizes linoleic acid to the toxic epoxides, leukotoxin and isoleukotoxin which have been implicated in multiple organ failure and acute respiratory distress (Spector and Kim, 2015). The ability of biochanin to permeate BBB is of tremendous advantage, as it suggests a therapeutic effect against glucose – neurotoxicity. The predicted high GI absorption of the compounds can be attributed to the lipophilic properties of flavonoids, making them able to cross the lipid membrane (Kumar and Pandey, 2013; Echeverría et al., 2017). The predicted oral lethal dose (LD_50_) and toxicity class of the compounds indicates its safety when orally ingested, this correlates with other predicted toxicity of isolated compounds from *C. volubile* leaves (Erukainure et al., 2017a,b,c).

## Conclusion

These results portray the therapeutic effect of *C. volubile* leaves against type 2 diabetes, with the DCM fraction being the most potent. The synergetic attenuation of pancreatic redox imbalance, dyslipidemia; modulation of glucose and insulin homeostasis; as well as improved pancreatic β-cell distribution and function suggests possible antidiabetic mechanisms. This is further evident by the molecular interaction of the isolated flavones from the DCM fraction with α-glucosidase *in silico*. Additionally, the molecular interaction of these flavones with ACE-1 and the observed inhibitory effect on this enzyme suggests antihypertensive potentials of this fraction, which can be further investigated in type 2 diabetic models. However, modulation of CYPs 1A2, 2C9, 2D6, and 3A4 by the flavones suggests caution in usage with other prescribed drugs metabolized by these enzymes. A further study is being proposed on the comparative antidiabetic properties of the isolated compounds with other standard antidiabetic drugs, particularly metformin and glibenclamide in type 2 diabetic models.

## Author Contributions

OE, RH, MC: designed the project. OE, RH, NK: performed antidiabetic experiments. OE and AM: performed Ca^2+^ level. OE, MC, and OA: isolated and elucidated the pure compounds. OA, PB, and RP: performed *in silico* studies. CC, MI, and EA: performed ACE inhibition. OE, RH, MI, PB, and CC wrote the manuscript. All authors revised the manuscript.

## Conflict of Interest Statement

The authors declare that the research was conducted in the absence of any commercial or financial relationships that could be construed as a potential conflict of interest.

## Supplementary Material

FIGURE S1 Acute effects of crude extract (MeOH), dichloromethane fraction (DCM), butanol fraction (BuOH) and aqueous fraction (aqueous) on blood glucose of diabetic rats. Values are means ±*SD* (*n* = 5). Control: non-diabetic control rats; Db: diabetic rats treated with saline only; Crude-200 mg/kg, and Crude-400 mg/kg: diabetic rats treated with MeOH extract of 200 and 400 mg/kg bw; DCM-200 mg/kg, and DCM-400 mg/kg: diabetic rats treated with DCM fraction of 200 and 400 mg/kg; BuOH-200 mg/kg, and BuOH-400 mg/kg: diabetic rats treated with BuOH fraction of 200 and 400 mg/kg bw; aqueous-200 mg/kg, and aqueous-400 mg/kg: diabetic rats treated with aqueous fraction of 200 and 400 mg/kg bw; GB-5mg/kg: diabetic rats treated with glibenclamide 5 mg/kg bw. ^∗^*p* < 0.05, ^∗∗^*p* < 0.01, ^∗∗∗^*p* < 0.001, compared to their respective 0-h values.

FIGURE S2 **(A)** Binding orientation and interaction of the original ligand lisinopril (in magenta) and the docked compounds 5,7,4′-trimethoxykaempferol (in blue) and Biochanin (in orange) into the active site of the ACE enzyme. **(B)** Ligand–protein interaction diagram: 5,7,4′-trimethoxykaempferol **(Left)** and Biochanin **(Right)** interacts with the protein ACE, respectively, via hydrogen bonds (shown in blue arrow) and π interactions (shown as orange lines) with the participating π systems and hydrophobic contacts.

FIGURE S3 Binding orientation and interaction of the original ligand acarbose (in magenta) and the docked compounds 5,7,4′-trimethoxykaempferol (in blue) and Biochanin (in orange) into the active site of the Human Maltase-Glucoamylase enzyme. **(B)** Ligand–protein interaction diagram: 5,7,4′-trimethoxykaempferol **(Left)** and Biochanin **(Right)** interacts with the protein Human Maltase-Glucoamylase, respectively, via hydrogen bonds (shown in blue arrow) and π interactions (shown as orange lines) with the participating π systems and hydrophobic contacts.
