# Supplementary figures and images for: Suppressive Effects of Clerodendrum volubile P Beauv. [Labiatae] Methanolic Extract and Its Fractions on Type 2 Diabetes and Its Complications
Source: Front Pharmacol. 2018 Feb 1;9:8. doi: 10.3389/fphar.2018.00008 (PMC5799276; doi:10.3389/fphar.2018.00008)

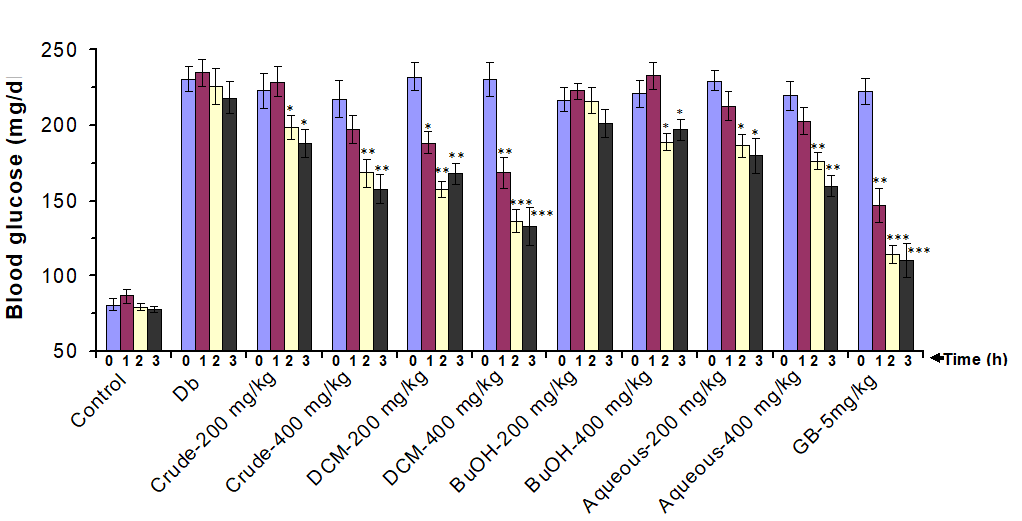

Supplement: FIGURE S1 — Acute effects of crude extract (MeOH), dichloromethane fraction (DCM), butanol fraction (BuOH) and aqueous fraction (aqueous) on blood glucose of diabetic rats. Values are means ±SD (n = 5). Control: non-diabetic control rats; Db: diabetic rats treated with saline only; Crude-200 mg/kg, and Crude-400 mg/kg: diabetic rats treated with MeOH extract of 200 and 400 mg/kg bw; DCM-200 mg/kg, and DCM-400 mg/kg: diabetic rats treated with DCM fraction of 200 and 400 mg/kg; BuOH-200 mg/kg, and BuOH-400 mg/kg: diabetic rats treated with BuOH fraction of 200 and 400 mg/kg bw; aqueous-200 mg/kg, and aqueous-400 mg/kg: diabetic rats treated with aqueous fraction of 200 and 400 mg/kg bw; GB-5mg/kg: diabetic rats treated with glibenclamide 5 mg/kg bw. ∗p < 0.05, ∗∗p < 0.01, ∗∗∗p < 0.001, compared to their respective 0-h values. [file Image_1.TIF]

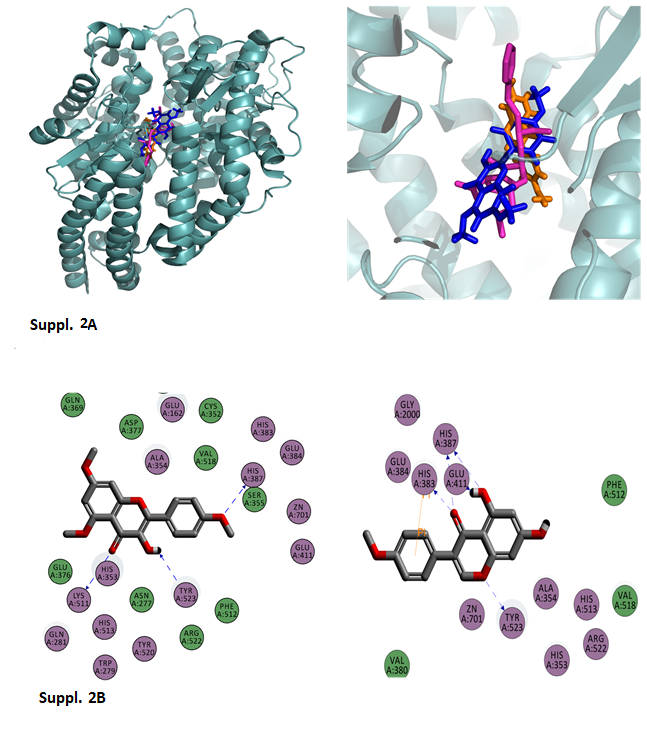

Supplement: FIGURE S2 — (A) Binding orientation and interaction of the original ligand lisinopril (in magenta) and the docked compounds 5,7,4′-trimethoxykaempferol (in blue) and Biochanin (in orange) into the active site of the ACE enzyme. (B) Ligand–protein interaction diagram: 5,7,4′-trimethoxykaempferol (Left) and Biochanin (Right) interacts with the protein ACE, respectively, via hydrogen bonds (shown in blue arrow) and π interactions (shown as orange lines) with the participating π systems and hydrophobic contacts. [file Image_2.tif]

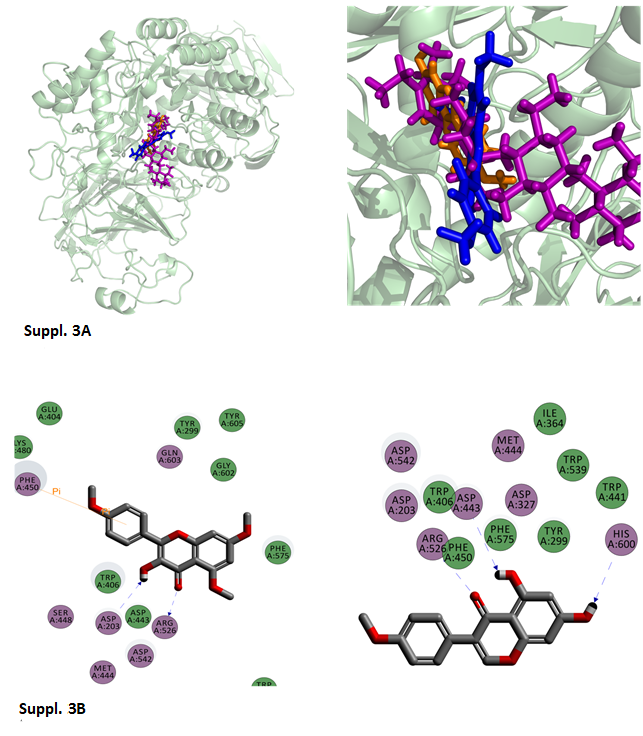

Supplement: FIGURE S3 — Binding orientation and interaction of the original ligand acarbose (in magenta) and the docked compounds 5,7,4′-trimethoxykaempferol (in blue) and Biochanin (in orange) into the active site of the Human Maltase-Glucoamylase enzyme. (B) Ligand–protein interaction diagram: 5,7,4′-trimethoxykaempferol (Left) and Biochanin (Right) interacts with the protein Human Maltase-Glucoamylase, respectively, via hydrogen bonds (shown in blue arrow) and π interactions (shown as orange lines) with the participating π systems and hydrophobic contacts. [file Image_3.tif]
